# Supplementary material for: Mastering preclinical proton and deuterium-based fast MRSI in the brain: from setup to execution
Source: MAGMA. 2026 Apr 8;39(3):395–416. doi: 10.1007/s10334-026-01352-9 (PMC13687300; doi:10.1007/s10334-026-01352-9)
Supplement: Supplementary file 1 [file 10334_2026_1352_MOESM1_ESM.docx]

**Mastering Preclinical Fast MRSI: From Setup to Execution**

Gianna Nossa^1,2,3,†,c^, Eloïse Mougel^1,2,3,†,c^, Brayan Alves^1,2,3^, Tan Toi Phan^1,2,3^, Alessio Siviglia^1,2,3^, Thi Ngoc Anh Dinh^1,2,3^, Thanh Phong Lê^1,2,3^ , Bernard Lanz^1,2,3^, Cristina Cudalbu^1,2,3^

*Affiliations*

^1^CIBM Center for Biomedical Imaging, Lausanne, Switzerland

^2^CIBM Pre-Clinical Imaging Section, École polytechnique fédérale de Lausanne (EPFL), Lausanne, Switzerland

^3^MRS4Brain Group, École polytechnique fédérale de Lausanne (EPFL), Lausanne, Switzerland

† This author contributed equally to this work

c. corresponding author:

Name: Gianna Nossa

email address: gianna.nossa@epfl.ch

telephone: +41 21 693 51 44

c. corresponding author:

Name: Eloïse Mougel

email address: eloise.mougel@epfl.ch

telephone: +41 21 693 68 48

**Supplemental Material**

**Supplemental Table 1** Minimum Reporting Standards in MRS for FID-MRSI at 9.4T and 14.1T

| **Hardware** | |
| --- | --- |
| **Field strength [T]** | 9.4T; 14.1T |
| **Manufacturer** | Bruker |
| **Model (software)** | 9.4T: Paravision 360 V3.5  14.1T: Paravision 360 V1.1 and V3.3 |
| **RF coil** | 9.4T: ^1^H-quadrature volume-transmit coil and a cryogenic four-channel receive rat head array  14.1T: ^1^H-quadrature surface rat head coil |
| **Additional Hardware** | N/A |

| **Acquisition** | |
| --- | --- |
| **Pulse Sequence** | FID-MRSI (Bruker:CSI) |
| **Volume of Interest (VOI)** | Rodent: Brain |
| **Nominal VOI Size** | 0.77 × 0.77 × 2 mm^3^ |
| **Repetition Time (TR) and Acquisition Delay (AD)** | 9.4T: TR = 813 ms / AD = 1.3 ms  14.1T: TR = 811.48 ms / AD = 1.3 ms |
| **Number of Averaged Spectra (NA)** | 1 average |
| **FOV in All Directions** | 24 × 24 × 2 mm^3^ FOV |
| **Matrix Size** | 31×31 |
| **Spectral Bandwidth (Hz)** | 9.4T: 5000  14.1T: 7142.85 |
| **Number of Acquired Spectral Points** | 9.4T: 768  14.1T: 1024 |
| **Water Suppression Method** | VAPOR |
| **Shimming Method** | Bruker MAPSHIM, first in an ellipsoid covering the full brain and further in a volume of interest centered on the MRSI slice, with a thickness of 2 mm |
| **Triggering or Motion Correction** | N/A |

| **Data Analysis** | |
| --- | --- |
| **Analysis Software** | LCmodel (Version 6.3-1N) |
| **Processing Step Parameters** | Custom Basis-Sets for AD = 1.3ms (for both 9.4T and 14.1T)  Control files provided with the *MRS4Brain Toolbox* |
| **Output Measure** | Ratios to total Creatine |
| **Quantification Reference** | Basis-set including: alanine, aspartate, ascorbate, creatine, phosphocreatine, γ-aminobutyrate, glutamine, glutamate, glycerophosphocholine, glutathione, glucose, inositol, N-acetylaspartate, N-acetylaspartylglutamate, phosphocholine, phosphoethanolamine, lactate, taurine simulated using NMR ScopeB. Macromolecules acquired in-vivo with double inversion recovery FID-MRSI. |

| **Data quality** | |
| --- | --- |
| **Reported Variables** | SNR (reference to NAA) and linewidths (reference to water) both reported  Global linewidths estimated by LCModel |
| **Data Exclusion Criteria** | LCModel SNR > 4 and  LCModel FWHM < 1.25*LCModel |
| **Quality Measures of Post-processing Model Fitting** | CRLB < 30% |
| **Sample Spectrum** | Figure 6 |

**Supplemental Table 2** MRSinMRS for PRESS-MRSI

| **Hardware** | |
| --- | --- |
| **Field Strength [T]** | 14.1 |
| **Manufacturer** | Bruker |
| **Model (software)** | Paravision 360 V3.3 |
| **RF coil** | ^1^H-quadrature surface rat head coil |
| **Additional Hardware** | N/A |

| **Acquisition** | |
| --- | --- |
| **Pulse Sequence** | PRESS-MRSI (Bruker:CSI) |
| **Volume of Interest (VOI)** | Rodent: Brain |
| **Nominal VOI Size** | 0.77 × 0.77 × 2 mm^3^ |
| **Repetition Time (TR) and Echo Time (TE)** | TR = 2000 ms / TE = 10.2 ms |
| **Number of Averaged Spectra (NA)** | 1 average |
| **FOV in All Directions** | 24 × 24 × 2 mm^3^ |
| **Matrix Size** | 31×31 |
| **Spectral Bandwidth (Hz)** | 7142.85 |
| **Number of Acquired Spectral Points** | 1024 |
| **Water Suppression Method** | VAPOR |
| **Shimming Method** | Bruker MAPSHIM, first in an ellipsoid covering the full brain and further in a volume of interest centered on the MRSI slice, with a thickness of 2 mm |
| **Triggering or Motion Correction** | N/A |

| **Data Analysis** | |
| --- | --- |
| **Analysis Software** | LCmodel (Version 6.3-1N) |
| **Processing Step Parameters** | Custom Basis-Set for TE = 10.2 ms  Control files provided with the *MRS4Brain Toolbox* |
| **Output Measure** | Ratios to total Creatine |
| **Quantification Reference** | Basis-set including: alanine, aspartate, ascorbate, creatine, phosphocreatine, γ-aminobutyrate, glutamine, glutamate, glycerophosphocholine, glutathione, glucose, inositol, N-acetylaspartate, N-acetylaspartylglutamate, phosphocholine, phosphoethanolamine, lactate, taurine simulated using NMR ScopeB. Macromolecules acquired in-vivo with double inversion recovery FID-MRSI. |

| **Data quality** | |
| --- | --- |
| **Reported Variables** | SNR (reference to NAA) and linewidths (reference to water) both reported  Global linewidths estimated by LCModel |
| **Data Exclusion Criteria** | LCModel SNR > 4 and  LCModel FWHM < 1.25*LCModel |
| **Quality Measures of Post-processing Model Fitting** | CRLB < 30% |
| **Sample Spectrum** | Figure 6 |

**Supplemental Table 3** Minimum Reporting Standards in MRS for SE-MRSI

| **Hardware** | |
| --- | --- |
| **Field strength [T]** | 9.4T |
| **Manufacturer** | Bruker |
| **Model (software)** | Paravision 360 V3.5 |
| **RF coil** | ^1^H-quadrature volume-transmit coil and a cryogenic four-channel receive rat head array |
| **Additional Hardware** | N/A |

| **Acquisition** | |
| --- | --- |
| **Pulse Sequence** | SE-MRSI (Bruker:CSI) |
| **Volume of Interest (VOI)** | Rodent: Brain |
| **Nominal VOI Size** | 0.77 × 0.77 × 2 mm^3^ |
| **Repetition Time (TR) and Echo Time (TE)** | TR = 2000 ms / TE = 3.284 ms |
| **Number of Averaged Spectra (NA)** | 1 average |
| **FOV in All Directions** | 24 × 24 × 2 mm^3^ |
| **Matrix Size** | 31×31 |
| **Spectral Bandwidth (Hz)** | 5000 |
| **Number of Acquired Spectral Points** | 768 |
| **Water Suppression Method** | VAPOR |
| **Shimming Method** | Bruker MAPSHIM, first in an ellipsoid covering the full brain and further in a volume of interest centered on the MRSI slice, with a thickness of 2 mm |
| **Triggering or Motion Correction** | N/A |

| **Data Analysis** | |
| --- | --- |
| **Analysis Software** | LCmodel (Version 6.3-1N) |
| **Processing Step Parameters** | Custom Basis-Sets for STEAM TE = 3 ms  Control files provided with the *MRS4Brain Toolbox* |
| **Output Measure** | Ratios to total Creatine |
| **Quantification reference** | Basis-set including: alanine, aspartate, ascorbate, creatine, phosphocreatine, γ-aminobutyrate, glutamine, glutamate, glycerophosphocholine, glutathione, glucose, inositol, N-acetylaspartate, N-acetylaspartylglutamate, phosphocholine, phosphoethanolamine, lactate, taurine simulated using NMR ScopeB. Macromolecules acquired in-vivo with double inversion recovery FID-MRSI. |

| **Data quality** | |
| --- | --- |
| **Reported Variables** | SNR (reference to NAA) and linewidths (reference to water) both reported  Global linewidths estimated by LCModel |
| **Data Exclusion Criteria** | LCModel SNR > 4 and  LCModel FWHM < 1.25*LCModel |
| **Quality Measures of Post-processing Model Fitting** | CRLB < 30% |
| **Sample Spectrum** | Figure 6 |

**Supplemental Table 4** MRSinMRS for SVS STEAM Metabolites and Water acquisitions used for quality assessment.

| **Hardware** | |
| --- | --- |
| **Field strength [T]** | 9.4T |
| **Manufacturer** | Bruker |
| **Model (software)** | 9.4T: Paravision 360 V3.5 |
| **RF coil** | ^1^H-quadrature volume-transmit coil and a cryogenic four-channel receive rat head array |
| **Additional Hardware** | N/A |

| **Acquisition** | |
| --- | --- |
| **Pulse Sequence** | Bruker: STEAM |
| **Volume of Interest (VOI)** | Rodent: Brain |
| **Nominal VOI Size** | 10 × 10 × 2 mm^3^ |
| **Repetition Time (TR), Echo Time (TE), Mixing Time (TM)** | TR = 4000 ms / TE = 3 ms / TM = 10 ms |
| **Number of Averaged Spectra (NA)** | 32 (metabolites); 16 (water) |
| **FOV in All Directions** | NA |
| **Matrix Size** | NA |
| **Spectral Bandwidth (Hz)** | 5000 |
| **Number of Acquired Spectral Points** | 4096 |
| **Water Suppression Method** | VAPOR (metabolites); NO SUPPRESSION (water) |
| **Shimming Method** | Bruker MAPSHIM, first in an ellipsoid covering the full brain and further in a volume of interest centered on the voxel |
| **Triggering or Motion Correction** | N/A |

| **Data Analysis** | |
| --- | --- |
| **Analysis Software** | LCmodel (Version 6.3-1N) |
| **Processing step Parameters** | Custom Basis-Sets for STEAM TE = 3 ms  Control files provided with the *MRS4Brain Toolbox* |
| **Output measure** | Ratios to total Creatine |
| **Quantification reference** | Basis-set including: alanine, aspartate, ascorbate, creatine, phosphocreatine, γ-aminobutyrate, glutamine, glutamate, glycerophosphocholine, glutathione, glucose, inositol, N-acetylaspartate, N-acetylaspartylglutamate, phosphocholine, phosphoethanolamine, lactate, taurine simulated using NMR ScopeB. Macromolecules acquired in-vivo with double inversion recovery FID-MRSI. |

| **Data quality** | |
| --- | --- |
| **Reported Variables** | SNR (reference to NAA) and linewidths (reference to water) both reported  Global linewidths estimated by LCModel |
| **Data Exclusion Criteria** | LCModel SNR > 4 and  LCModel FWHM < 1.25*LCModel |
| **Quality Measures of Post-processing Model Fitting** | CRLB < 30% |
| **Sample Spectrum** | Figure 6 |
